# Triceps Motor Branch Transfers to the Infraspinatus and Teres Minor Motor Branches for Restoration of Shoulder External Rotation: A Cadaver Study

**DOI:** 10.1002/micr.70289

**Published:** 2026-09-13

**Authors:** Bruno Azevedo Veronesi, Ângelo Santana Guerra, Renata Gregorio Paulos, Hugo Alberto Nakamoto, Teng Hsiang Wei, Marcelo Rosa de Rezende

**Affiliations:** ^1^ Department of Hand Surgery and Microsurgery Institute of Orthopedics and Traumatology of the Clinics Hospital, School of Medicine, University of São Paulo (IOT‐HCFMUSP) São Paulo São Paulo Brazil

**Keywords:** brachial plexus, nerve transfer, peripheral nerve injuries, radial nerve, shoulder joint/innervation

## Abstract

**Background:**

Recovery of shoulder external rotation after upper brachial plexus injury remains limited. This study investigated the anatomical feasibility of selective neurotizations using radial nerve branches to the triceps brachii as donors for the motor branches to the infraspinatus (bIS) and teres minor (bTm) muscles, aiming to enhance external rotation outcomes.

**Methods:**

Twenty fresh adult cadavers underwent posterior dissections to expose the suprascapular, axillary, and radial nerves. Radial nerve branches to the triceps were identified, measured, and mobilized to simulate transfers to bIS and bTm. Donor–recipient lengths, diameters, and feasibility of coaptation were analyzed. In 10 cadavers, histomorphometric evaluation quantified myelinated axons to assess compatibility.

**Results:**

The most frequent branching pattern of the radial nerve included one branch to the long head of the triceps (bLoHT), two branches to the medial head (bMHT1 and bMHT2), and one or two branches to the lateral head (bLaHT1 and bLaHT2). bMHT2 was the longest branch (93.0 ± 8.0 mm, *p* < 0.05), showed the highest axon count (3646 ± 2333 axons, *p* < 0.05), and had the best reach to bIS. bLoHT and bMHT2 were the thickest branches (1.99 ± 0.49 mm and 2.07 ± 0.58 mm, respectively; *p* < 0.05). The most feasible transfers were bMHT2 to bIS and bMHT1 to bTm, both demonstrating 100% feasibility, with diameter and axon count measurements also supporting these neurotizations.

**Conclusions:**

Selective transfers from triceps branches to bIS and bTm are anatomically feasible. Medial head branches combine favorable length, diameter, and axon load, supporting their use as preferred donors to restore external rotation.

## 1 Introduction

Injuries of the brachial plexus that specifically involve the C5–C6 roots or the upper trunk cause deficits in shoulder abduction and external rotation, as well as elbow flexion. The traditional treatment for these patients consists of reconstruction with nerve grafts, but this is not an option when there is root avulsion, and it usually yields poor functional results when the approach is delayed or when the regeneration distance is long (Rinker 2015). In such cases, nerve transfer or neurotization is considered the best surgical option.

The most common nerve transfers for upper brachial plexus injuries include the ulnar fascicles to the biceps branch, the spinal accessory to the suprascapular nerve (SSN), and radial nerve branches to the axillary nerve (Bertelli and Ghizoni 2010). This “triple neurotization” generally provides better results than nerve grafting; however, recovery of shoulder external rotation remains the most limited (Garg et al. 2011). Many patients regain elbow flexion yet maintain a “hand‐on‐belly” posture that restricts limb function.

The spinal accessory to suprascapular transfer frequently fails to reinnervate the infraspinatus, possibly due to preferential axonal regeneration toward the supraspinatus or a double‐level traction injury of the SSN (Vekris et al. 2010). In addition, transfers of a radial nerve branch to the axillary nerve often reinnervate only its anterior division, restoring the deltoid but not the teres minor, which receives its branch from the posterior division (Witoonchart et al. 2003).

As the infraspinatus and teres minor act synergistically as the main external rotators of the shoulder (Williams et al. 2018), selective reinnervation of both may enhance torque and overall shoulder function. Recent anatomical studies have proposed isolated transfers to one of these muscles (Tavares et al. 2020; Kaiser et al. 2021; Krajcová et al. 2023), but none have simultaneously targeted both in adults.

**FIGURE 1:**
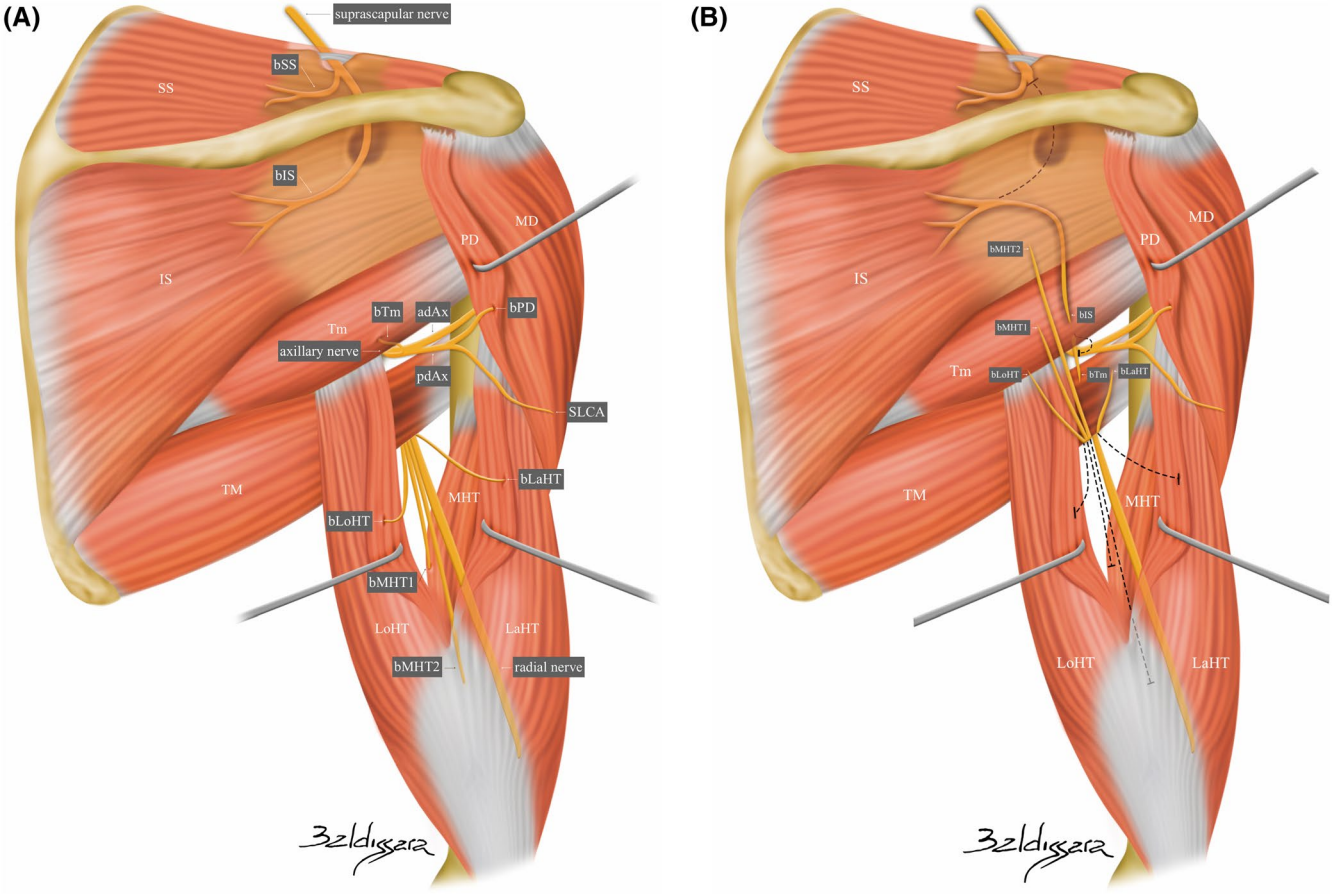
(A) Anatomy of the suprascapular, axillary, and radial nerves. (B) Mobilization of the motor branches of the triceps brachii and the motor branches of the infraspinatus and teres minor to evaluate the viability of the neurotizations. adAx, anterior division of the axillary nerve; bIS, motor branch to the infraspinatus; bLaHT, motor branch to the lateral head of the triceps; bLoHT, motor branch to the long head of the triceps; bMHT, motor branches to the medial head of the triceps (bMHT1, upper branch; bMHT2, lower branch); bPD, motor branch to the posterior deltoid; bSS, motor branch to the supraspinatus; bTm, motor branch to the teres minor; IS, infraspinatus; LaHT, lateral head of the triceps; LoHT, long head of the triceps; MD, middle deltoid; MHT, medial head of the triceps; PD, posterior deltoid; pdAx, posterior division of the axillary nerve; SLCA, superior lateral cutaneous nerve of the arm; SS, supraspinatus; Tm, teres minor; TM, teres major.

The present cadaveric study aimed to evaluate the anatomical feasibility of selective posterior transfers of radial nerve branches to the triceps as donors for the motor branches to the infraspinatus (bIS) and teres minor (bTm) (Figure 1). Distances, diameters, and axon counts were analyzed to identify the most suitable donor–recipient combinations for selective reinnervation of shoulder external rotators.

## 2 Materials and Methods

This anatomical observational study was conducted on 20 fresh adult cadavers, totaling 20 dissections, all performed on the right side for standardization. None of the specimens presented previous trauma, surgery, or deformity in the shoulder region. The study followed institutional ethical guidelines and complied with the Anatomical Quality Assurance (AQUA) checklist to ensure methodological consistency and reproducibility (Tomaszewski et al. 2017).

### 2.1 Cadaveric Dissections

The dissections were performed through a posterior approach with cadavers in the prone position, the shoulder abducted at 20°, and the elbow extended. A posterior incision was made parallel and 2 cm cranial to the scapular spine and, near the acromial angle, extended along the proximal two‐thirds of the posterior midline of the arm.

The posterior deltoid was detached from the scapular spine and retracted laterally to expose the infraspinatus and teres minor. In the inferior portion of the posterior deltoid, the superior lateral cutaneous nerve of the arm was identified and traced to its origin from the posterior division of the axillary nerve. This nerve was dissected through the quadrangular space, where the anterior and posterior divisions of the axillary nerve were identified, and the motor branches to the teres minor and posterior deltoid were exposed.

In the posterior arm, blunt dissection between the long and lateral triceps heads revealed the medial head. At the inferior border of the teres major, the radial nerve and its branches to the triceps were exposed and followed from their origin in the main trunk to their muscular entries.

The dissection then progressed toward the scapular region. The infraspinatus was detached from the lateral scapular spine, where it was loosely adherent to bone. Its motor branch was identified after passing through the spinoglenoid notch and followed to its muscular entry. Partial detachment of the trapezius from the scapular spine exposed the supraspinatus. The plane between the supraspinatus and the scapula was developed, allowing visualization of the SSN beneath the superior transverse scapular ligament at the suprascapular notch; the ligament was sectioned. After identifying the supraspinatus branch, the SSN was traced through the spinoglenoid notch.

**FIGURE 2:**
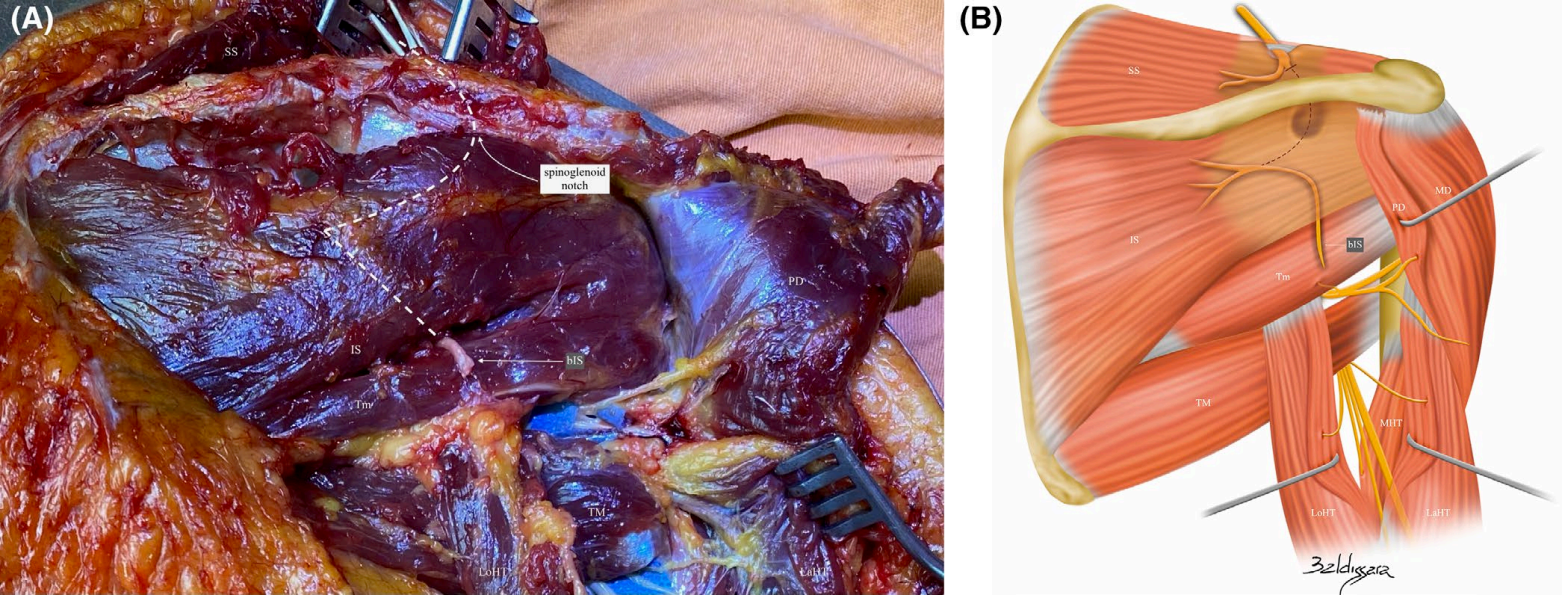
Photograph (A) and Illustration (B) of the motor branch of the infraspinatus mobilization to the interval between the infraspinatus and teres minor muscles. bIS, motor branch to the infraspinatus; IS, Infraspinatus; LaHT, lateral head of the triceps; LoHT, Long head of the triceps; MD, Middle deltoid; MHT, medial head of the triceps; PD, posterior deltoid; SS, supraspinatus; Tm, teres minor; TM, teres major.

To simulate transfers, recipient branches were sectioned at their origin from the main trunks and donor branches at their muscular entries. The bIS was divided after the supraspinatus branch, distal to the superior transverse scapular ligament, and mobilized into the infraspinatus fossa through the spinoglenoid notch toward the interval between the infraspinatus and teres minor, as proposed by Wyles et al. (2019) (Figure 2).

### 2.2 Measurements and Feasibility Analysis

**FIGURE 3:**
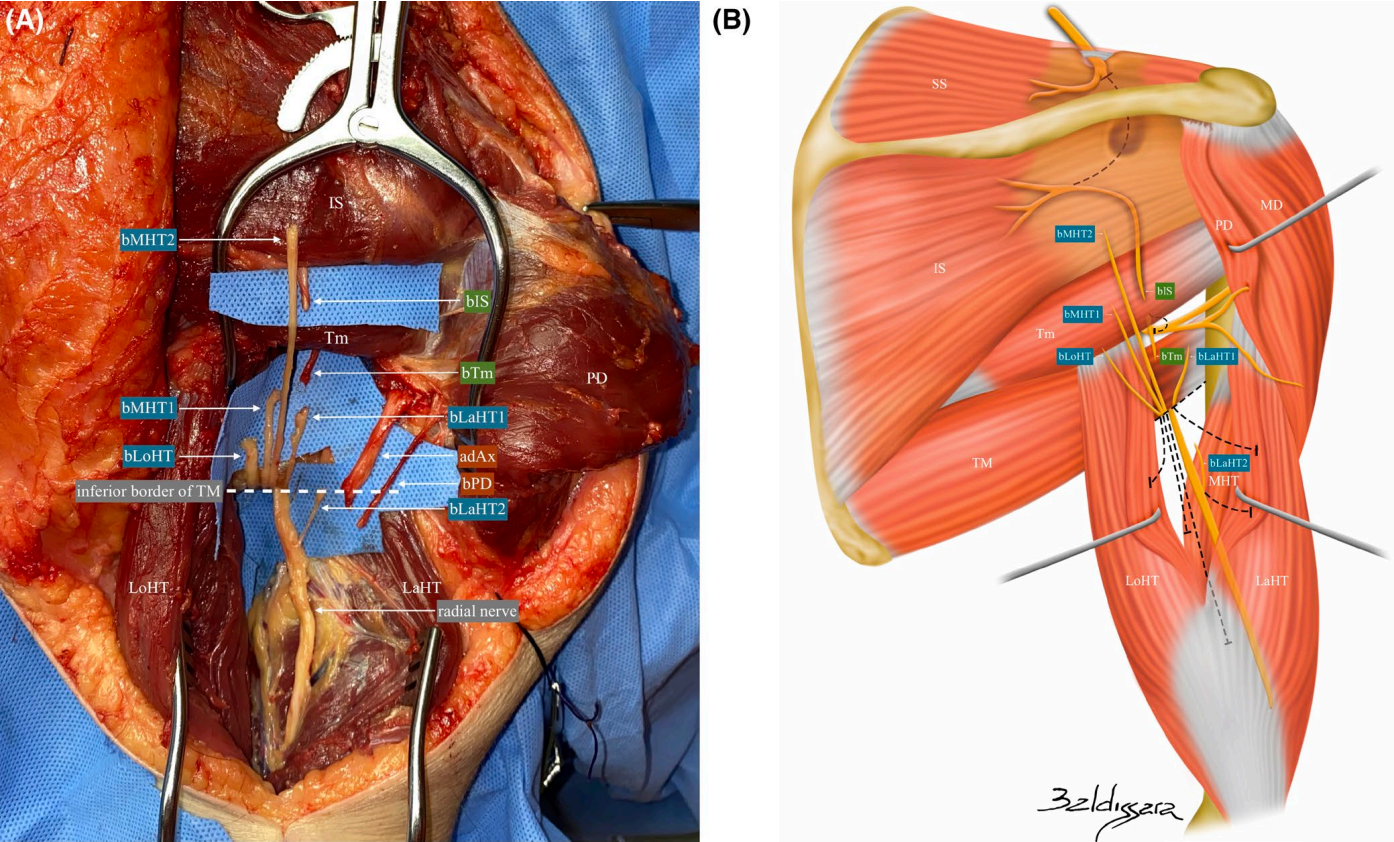
Photograph (A) and illustration (B) of nerve positioning to simulate neurotization between the radial nerve branches to the triceps brachii and the infraspinatus and teres minor branches. adAx, anterior division of the axillary nerve; bIS, motor branch to the infraspinatus; bLaHT, motor branches to the lateral head of the triceps (bLaHT1, upper branch; bLaHT2, Lower branch); bLoHT, motor branch to the long head of the triceps; bMHT, motor branches to the medial head of the triceps (bMHT1, upper branch; bMHT2, lower branch); bPD, motor branch to the posterior deltoid; bTm, motor branch to the teres minor; IS, infraspinatus; LaHT, lateral head of the triceps; LoHT, long head of the triceps; MD, middle deltoid; MHT, Medial head of the triceps; PD, posterior deltoid; SS, supraspinatus; Tm, teres minor; TM, Teres major.

The measurements were taken using a precision digital caliper (resolution 0.01 mm; precision ±0.02 mm). The lengths of the motor branches—recipients: bIS, bTm; and donors: bLoHT, bLaHT1–2, and bMHT1–2—were recorded. After mobilizing the branches toward the potential neurotizations, the positions of their free ends in relation to the lower edge of the teres major muscle were measured, with positive values indicating points above and negative values below this line (Figure 3). This reference (lower edge of the teres major muscle) was used because it represents the anatomical shield through which the triceps branches—potential donor nerves—redirect from distal to proximal in the arm toward the recipient nerves.

Because nerves and muscles present elasticity and are not collinear in their course, each transfer between donor and recipient branches was simulated to confirm whether coaptation was possible without nerve tension. When feasible, the overlap interval between donor and recipient nerves was recorded. The diameters of these branches were also measured at their free ends after neurotomies.

### 2.3 Histomorphometric Analysis

In this same region, in 10 cadavers whose families had provided consent for the removal of nerve fragments, samples measuring 1 cm in length were collected from each branch of the studied nerves. The samples were fixed in 4% paraformaldehyde and then in a solution of osmium tetroxide and ferricyanide. Dehydration was performed with acetone solutions. After embedding in Epon resin, polymerization was carried out. Semithin sections were obtained using an ultramicrotome and a glass knife. These sections were heat‐fixed, stained with 1% methylene blue, washed with sterile distilled water, and dried. Histological processing was performed at the Electron Microscopy Laboratory of the Adolfo Lutz Institute by an experienced research team. The slides were examined under a Carl Zeiss microscope (Axiolab model) equipped with a JVC TK‐1270 camera, and digital photographs were obtained at ×400 magnification. Myelinated fibers were manually counted by a single investigator (one of the authors) using digital images and a point‐marking method to avoid double counting.

### 2.4 Statistical Analysis

Continuous variables are presented as mean ± standard deviation (SD), 95% confidence interval (CI), and minimum and maximum values, while categorical data are expressed as *n* (%). The normality of continuous variables was tested using the Shapiro–Wilk test. For normally distributed data, comparisons between two groups were performed with Student's *t*‐test for paired samples, and among three or more groups using repeated‐measures ANOVA with Bonferroni correction. For nonnormally distributed data, the Wilcoxon test was used for two groups and the Friedman test for three or more. Correlations were assessed using Pearson's coefficient for normally distributed data and Spearman's coefficient for nonnormal data. *p* ≤ 0.05 was considered statistically significant.

## 3 Results

Of the 20 cadavers, 13 were male (65%) and seven were female (35%). The mean age was 67.8 ± 13.1 years, mean height 173.2 ± 10.4 cm, and mean weight 60.7 ± 17.6 kg.

In all dissections, a single branch arising from the main trunk of the radial nerve to the long head of the triceps (bLoHT) was identified. In 19 dissections (95%), two branches to the medial head of the triceps (bMHT1 and bMHT2) were observed, whereas in one case only a single branch was present, traveling to the distal region of the muscle (bMHT2). The lateral head received one branch in nine cadavers (45%) and two branches in the remaining 11 (55%) (bLaHT1 and bLaHT2). Among the cases with two branches, seven had the inferior branch (bLaHT2) originating from the inferior branch to the medial head (bMHT2). Thus, the most frequent pattern consisted of one branch to the long head, two branches to the medial head, and, with greater variation, two branches to the lateral head—the inferior branch arising from bMHT2.

The first branch from the radial nerve to the triceps brachii was consistently the bLoHT, located near the lower border of the teres major muscle, followed by bMHT1, bMHT2, and then the branches to the lateral head (bLaHT1 and bLaHT2). The bLoHT and bLaHT1 ran obliquely in opposite directions toward the proximal portions of the long and lateral heads of the triceps, respectively. The bMHT1 consistently passed medial to the radial nerve, entering the medial head proximally, whereas bMHT2 lay superficial to the radial nerve and then continued juxtamedial and parallel to it until its distal muscular entry, near the radial groove.

The lengths and diameters of all branches are summarized in Table 1. Among the triceps branches, bMHT2 was the longest (93.0 ± 8.0 mm; *p* < 0.05), and both bMHT2 and bLoHT had the greatest diameters (2.07 ± 0.58 mm and 1.99 ± 0.49 mm, respectively; *p* < 0.05). When comparing donor and recipient branches, bIS exhibited a larger diameter than the lateral head branches and bMHT1, but no significant difference compared to bLoHT and bMHT2. The bTm had a greater diameter than the lateral head branches but was similar in size to bLoHT and the medial head branches.

**TABLE 1:** Branch lengths and diameters.

Motor branch	*n*	Length, mean ± SD (mm)	95% CI (mm)	Diameter, mean ± SD (mm)	95% CI (mm)
IS	20	40.8 ± 7.4	37.3–44.3	2.05 ± 0.41	1.86–2.25
Tm	20	22.6 ± 4.9	20.3–24.9	1.95 ± 0.53	1.70–2.20
LoHT	20	37.8 ± 10.7	32.8–42.8	1.99 ± 0.49	1.77–2.22
LaHT1	19	41.2 ± 10.3	36.2–46.1	1.24 ± 0.30	1.10–1.39
LaHT2	12	40.5 ± 15.8	30.5–50.5	0.86 ± 0.34	0.64–1.08
MHT1	19	54.3 ± 12.2	48.4–60.2	1.56 ± 0.34	1.40–1.72
MHT2	20	93.0 ± 8.0	89.3–96.7	2.07 ± 0.58	1.80–2.34

**FIGURE 4:**
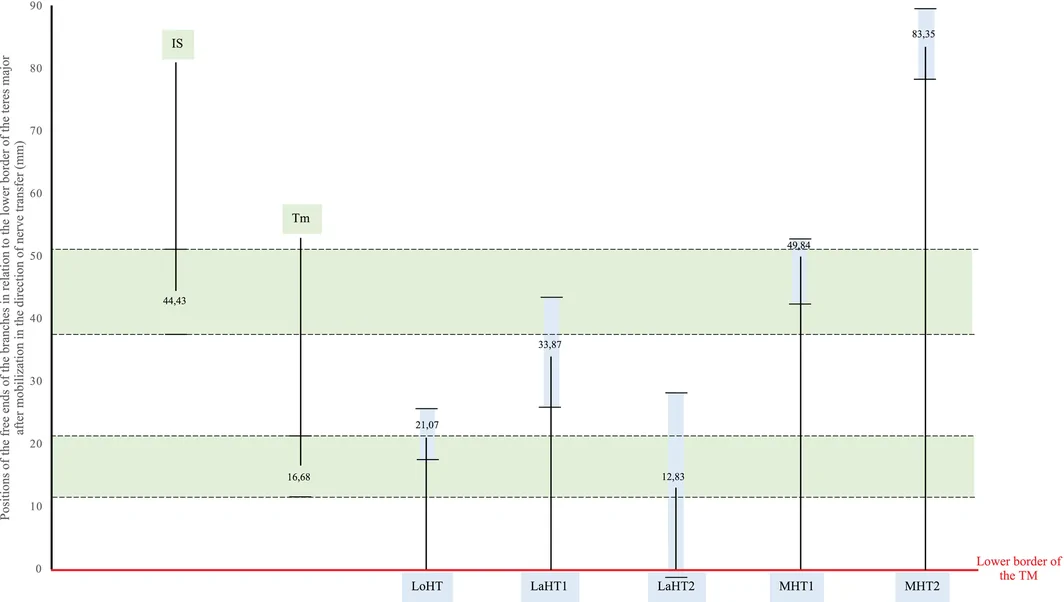
Positions of the free ends of each branch in relation to the lower border of the teres major muscle after mobilization in the direction of nerve transfer (mean and 95% CI).

The mean positions and 95% CIs of the free ends of each branch relative to the lower border of the teres major muscle after mobilization toward nerve transfers are illustrated in Figure 4. Based on this indirect evaluation, considering the means and 95% CIs, the triceps branch with the greatest reach to bIS was bMHT2, while for bTm, bLaHT1 and both medial head branches demonstrated the best proximity.

A comparative analysis of the relative positions of the triceps branches confirmed that bMHT2 provided the longest reach to the recipient branches, followed by bMHT1 (*p* < 0.05). Considerable variation in the position of bLaHT2 was observed due to its variable origin, either from the main trunk or from bMHT2.

The feasibility of nerve transfers in the simulations, as well as the overlap intervals between branches in viable cases, are presented in Table 2. The most feasible transfers to bIS and bTm were from bMHT2 and bMHT1, respectively, both demonstrating 100% feasibility, with mean overlaps of 37.8 ± 19.8 mm and 33.4 ± 21.5 mm, respectively.

**TABLE 2:** Feasibility of nerve transfers and overlap between donors and recipients.

Motor branches	Feasibility	Overlap (mean ± SD) (mm)	Minimum (mm)	Maximum (mm)
IS‐LoHT	2/20 (10%)	17.5 ± 24.7	0	34.91
IS‐LaHT1	6/19 (31.6%)	15.7 ± 30.3	0	77.13
IS‐LaHT2	2/12 (16.7%)	0 ± 0	0	0
IS‐MHT1	16/19 (84.2%)	14 ± 18.1	0	54.55
IS‐MHT2	20/20 (100%)	37.8 ± 19.8	9.02	94.48
Tm‐LoHT	16/20 (80%)	12.7 ± 10.1	0	34.35
Tm‐LaHT1	16/19 (84.2%)	16.6 ± 17.9	0	72.13
Tm‐LaHT2	4/12 (33.3%)	28.9 ± 5.3	22.50	35.23
Tm‐MHT1	19/19 (100%)	33.4 ± 21.5	0	74.36
Tm‐MHT2	20/20 (100%)	60.5 ± 19.7	16.03	96.93

For the histological analysis (Table 3), 21% of slides, with poor‐quality photographs unsuitable for axon counting, were excluded. Among the triceps branches, bMHT2 had the highest axon count (3646 ± 2333 axons; *p* < 0.05). Comparing donor and recipient branches, bIS showed significantly more axons (3221 ± 924 axons) than all triceps branches, except for bMHT2 (*p* < 0.05). The bTm had a significantly higher axon count than bLoHT (1877 ± 556 vs. 1411 ± 418 axons), but there were no statistically significant differences when compared with the other triceps branches (*p* < 0.05). These results should be interpreted with caution due to the small sample size and variability in axon counts.

**TABLE 3:** Axon counts and proportions between the numbers of axons in donor and recipient nerves.

Motor branch	*n*	Mean ± SD	Minimum	Maximum
IS	9	3221 ± 924	2167	5001
Tm	7	1877 ± 556	1046	2601
LoHT	8	1411 ± 418	735	2049
LaHT1	5	989 ± 798	198	2117
LaHT2	3	371 ± 176	177	521
MHT1	8	1185 ± 317	687	1631
MHT2	9	3646 ± 2333	1541	8160
LoHT/IS	7	46% ± 9%	30%	55%
LaHT1/IS	5	34% ± 30%	6%	71%
LaHT2/IS	3	11% ± 6%	4%	16%
MHT1/IS	7	41% ± 17%	21%	68%
MHT2/IS	8	130% ± 98%	45%	282%
LoHT/Tm	5	82% ± 9%	70%	92%
LaHT1/Tm	4	52% ± 62%	9%	144%
LaHT2/Tm	2	20% ± 16%	9%	31%
MHT1/Tm	5	83% ± 45%	32%	156%
MHT2/Tm	6	170% ± 88%	92%	323%

**FIGURE 5:**
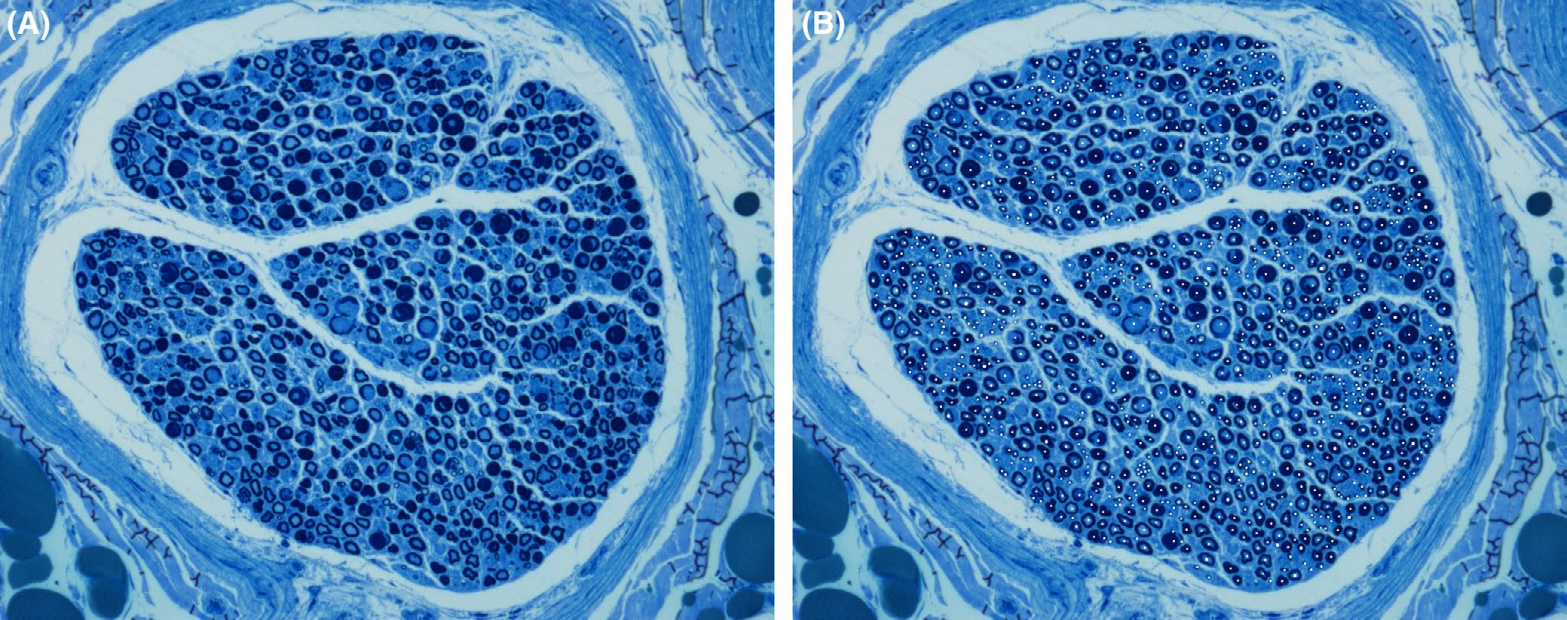
Representative slide photograph (×400 magnification) before (A) and after (B) counting of myelinated fibers.

Figure 5 shows a representative histological image before and after axon counting.

## 4 Discussion

For functional recovery of shoulder movement in upper brachial plexus injuries, double neurotization of the suprascapular and axillary nerves yields better external‐rotation outcomes than single transfer (Estrella et al. 2023). We evaluated the feasibility of selective neurotizations directed specifically to the motor branches of these nerves that supply the infraspinatus and teres minor muscles.

Our findings support the strategy of Wyles et al. (2019) to section the SSN distal to the supraspinatus branch, after its passage beneath the superior transverse scapular ligament. This approach increases the reach of the bIS and mitigates two theoretical drawbacks of spinal accessory‐to‐suprascapular transfers: concomitant injury at the suprascapular notch and preferential reinnervation of the supraspinatus. In selective transfers to the bIS, any excess length should preferably be shortened on the recipient rather than on the donor, bringing the coaptation closer to the neuromuscular junction and possibly avoiding inclusion of the segment that traverses the spinoglenoid notch, another potential site of injury.

The teres minor muscle is also a relevant target. Although the smallest rotator cuff muscle, it contributes approximately 25% of the glenohumeral external rotation torque, while the supraspinatus and infraspinatus together contribute about 75% (Kuhlman et al. 1992). Even modest gains in external rotation can improve function in brachial plexus injury (Langer et al. 2012). Moreover, the teres minor can hypertrophy and compensate when the supraspinatus and infraspinatus are nonfunctional, reinforcing its suitability for selective reinnervation (Kikukawa et al. 2014).

The branching pattern of the radial nerve identified in this study was consistent with previous anatomical descriptions (Bertelli et al. 2007), comprising one branch to the long head, two to the medial head, and variable branching—one or two—to the lateral head, with the inferior branch frequently originating from bMHT2. On average, the longest branches were those destined for the medial head of the triceps, confirming its suitability as a donor in nerve transfer procedures.

When assessing the viability and compatibility of nerve transfers, three main parameters were considered: adequate branch length to allow coaptation, sufficient axonal load to ensure reinnervation, and compatible nerve diameters. Indirect feasibility was measured in reference to the lower border of the teres major, as described by Khair et al. (2016), while direct simulations were performed for each potential transfer. The simulations generally demonstrated slightly greater feasibility than the indirect assessments, likely due to the elasticity of muscles and nerves, which allowed tension‐free approximation of donor and recipient branches when the distances were minimal. This phenomenon explains the “zero” overlap values observed in some measurements.

Our results indicated that the most favorable combinations for external rotation reconstruction—with the greatest likelihood of success—were bMHT2 to bIS and bMHT1 to bTm. Sectioning part of the teres major fascia, as suggested by Leechavengvongs et al. (2003) and Colbert and Mackinnon (2006), can increase the reach of the triceps branches to the recipient nerves, expand the options for neurotization, and possibly cause a slight weakening of adduction and internal rotation—movements opposite to those intended to be restored after surgery. This fascial release also allows the donor branch to course over a muscular rather than a fascial plane.

Diameter comparisons favored recipient pairings with bLoHT and medial‐head branches, all averaging close to 2 mm and consistent with prior studies (Tavares et al. 2020; Krajcová et al. 2023).

For histomorphometry, manual counting of myelinated axons was used to avoid sampling errors common in semiautomated methods, which may over‐ or underestimate axon density due to uneven fiber distribution (Geuna et al. 2001). This technique is simple, cost‐effective, and requires no specialized software.

Marked variability was observed in axon counts among cadavers, with large SDs, consistent with previous reports (Khair et al. 2016; Cheah et al. 2019). For instance, Khair et al. (2016) reported wide ranges for triceps branches—553–2771 for the long head, 679–4428 for the medial head, and 211–4187 for the lateral head. Across studies analyzing axon counts of the infraspinatus, teres minor, and triceps branches, results vary substantially between them (Witoonchart et al. 2003; Tavares et al. 2020; Khair et al. 2016). As emphasized by Cheah et al. (2019), intra‐study ratios between donor and recipient axon counts are more meaningful than absolute values or inter‐study comparisons, given the heterogeneity of counting methods.

Regarding axon ratios, there is no consensus on the minimum donor‐to‐recipient proportion required for satisfactory outcomes (Khair et al. 2016; Cheah et al. 2019; Schreiber et al. 2015). The classic rat study by Tötösy de Zepetnek et al. (1992) suggested ≥ 30% of original motor fibers could restore normal strength, but it is not directly generalizable to human transfers. Clinically, Schreiber et al. (2015) correlated ratios with elbow‐flexion recovery and suggested optimization when ≥ 70%. In our series, considering average ratios ≥ 70% and minima > 30%, the most favorable matches were bIS–bMHT2 and bTm–bLoHT or –bMHT1; however, variability in axon counts and sample size warrant cautious interpretation.

The spinal accessory nerve (SAN) to SSN transfer remains the most widely used technique for restoring shoulder external rotation. Histomorphometric studies report that the SAN contains approximately 863–1671 myelinated axons, whereas the SSN contains approximately 3500–6004 axons (Bhandari and Deb 2011; Pruksakorn et al. 2007), indicating a donor‐to‐recipient mismatch. Recovery of external rotation with this technique has been inconsistent in the literature. In the present study, the motor branches of the medial head of the triceps demonstrated axon counts ranging from 1185 to 3646, and the transfer was performed selectively to the infraspinatus branch of the SSN, which showed a mean axon count of 3221. This approach may provide a more favorable axonal match and enable more direct and selective reinnervation.

Notably, successful clinical outcomes have been reported even in nerve transfers that do not fully meet the theoretical requirements for compatibility in axon counts or diameters particularly in more distal transfers where targets are smaller and require fewer motor fibers (Cheah et al. 2019).

There is no consensus regarding how many triceps branches can be safely used as donors for nerve transfers. Several authors have reported preserved elbow extension strength after using a single triceps head as a donor (Leechavengvongs et al. 2003; Bertelli and Ghizoni 2004). Supporting the feasibility of using multiple triceps branches as donors—including concomitant branches from two heads—is the good recovery of elbow extension observed in patients with tetraplegia who underwent neurotization of only one triceps branch (Bertelli et al. 2011).

Similarly, no definitive agreement exists regarding which triceps branch is best suited as a donor for nerve transfers. Our findings favor the use of the medial head branches. Krauss et al. (2022) reported normal postoperative triceps strength in all patients who underwent transfer of the medial head branch to the axillary nerve. They advocated this branch because it is long, thick, and easily accessible without requiring intramuscular dissection—features confirmed in our dissections. Moreover, using this branch preserves the long head, which contributes to scapulohumeral adduction and elbow extension—movements that complement the function of the paralyzed posterior deltoid. Other series also supported the use of the medial head branches as donors (Tavares et al. 2020; Khair et al. 2016; Ellabban et al. 2021).

Based on the literature and the findings of this study, a potential clinical strategy using a posterior approach may include selective transfers: distal SAN to the supraspinatus branch, bMHT2 to the bIS, and bMHT1, with or without a lateral head triceps branch, to the bTm and adAx. When an additional LaHT branch is not available, the inferior subscapular nerve may be considered as an alternative donor.

The main limitation of this study is the relatively small number of dissected cadavers (*n* = 20). The sample size was determined by the availability of fresh specimens and was comparable to that of previous anatomical studies with similar objectives (Krajcová et al. 2023; Wyles et al. 2019; Bertelli et al. 2007). Histomorphometric analysis was performed in 10 cadavers, a number consistent with other investigations on nerve axon quantification (Witoonchart et al. 2003; Tavares et al. 2020; Khair et al. 2016; Cheah et al. 2019; Schreiber et al. 2015). However, approximately 21% of the slides were excluded because of inadequate image quality for axon counting, which may have reduced the statistical power of the analysis. Additionally, in patients with higher BMI, surgical exposure may be more challenging, particularly within the quadrilateral space due to the deeper location of the axillary nerve and its branches, although identification of the superior lateral cutaneous nerve of the arm may help guide dissection.

## 5 Conclusion

The transfer of radial nerve branches to the infraspinatus and teres minor muscles proved to be anatomically feasible. Among the potential donors, the branches to the medial head of the triceps demonstrated the most favorable parameters—length, diameter, and axon count—showing the greatest potential for successful reinnervation. The combinations bMHT2–bIS and bMHT1–bTm were the most reliable, supporting the use of medial head branches as preferred donors for selective neurotizations aimed at restoring shoulder external rotation.

## Author Contributions

Each author contributed individually and significantly to the development of this article: **Bruno Azevedo Veronesi:** writing – original draft, review and editing, conceptualization, methodology, investigation, data curation, visualization, formal analysis, and project administration. **Ângelo Santana Guerra:** writing – original draft, conceptualization, methodology, investigation, visualization, and formal analysis. **Renata Gregorio Paulos:** writing – original draft, conceptualization, methodology, investigation, and visualization. **Hugo Alberto Nakamoto:** writing – original draft, conceptualization, methodology, investigation, and visualization. **Teng Hsiang Wei** and **Marcelo Rosa de Rezende:** writing – review and editing, conceptualization, methodology, and investigation.

## Funding

The authors have nothing to report.

## Ethics Statement

This study was approved by the Research Ethics Committee of the Hospital das Clínicas, Faculdade de Medicina, Universidade de São Paulo (HCFMUSP), Brazil (CAAE number: 30156519.3.0000.0068; Approval No. 3.974.694). The study was conducted in accordance with institutional ethical guidelines.

## Consent

The families of ten cadavers signed the informed consent form for the removal of nerve fragments.

## Conflicts of Interest

The authors declare no conflicts of interest.

## Data Availability

The data that support the findings of this study are available from the corresponding author upon reasonable request.
